# Recent Progress in Chemical Composition, Production, and Pharmaceutical Effects of Kombucha Beverage: A Complementary and Alternative Medicine

**DOI:** 10.1155/2020/4397543

**Published:** 2020-11-18

**Authors:** Seyyed Mojtaba Mousavi, Seyyed Alireza Hashemi, Maryam Zarei, Ahmad Gholami, Chin Wei Lai, Wei Hung Chiang, Navid Omidifar, Sonia Bahrani, Sargol Mazraedoost

**Affiliations:** ^1^Biotechnology Research Center, Shiraz University of Medical Sciences, Shiraz, Iran; ^2^Department of Chemical Engineering, National Taiwan University of Science and Technology, Taipei, Taiwan; ^3^Department of Medical Nanotechnology, School of Advanced Medical Sciences and Technologies, Shiraz University of Medical Sciences, Shiraz, Iran; ^4^Pharmaceutical Sciences Research Center, Shiraz University of Medical Science, Shiraz, Iran; ^5^Nanotechnology & Catalysis Research Centre, University of Malaya, Kuala Lumpur, Malaysia; ^6^Clinical Education Research Center, and Pathology Department, Medical School, Shiraz University of Medical Sciences, Shiraz, Iran

## Abstract

Kombucha is a valuable traditional natural tea that contains beneficial compounds like organic acids, minerals, different vitamins, proteins, polyphenols, and several anions. Kombucha possesses anticancer, antioxidant, antimicrobial, and antifungal activity as well as hepatoprotective effects. Considering the unique properties of Kombucha, several investigations have already been conducted on its nutritional properties. In this review, an effort has been devoted to pool recent literature on the biomedical application of Kombucha under the objectives, including the chemical composition of Kombucha and industrial production, and highlight different properties of Kombucha. Finally, we explain its adverse effects and prospect. This review is an active, in-depth, and inclusive report about Kombucha and its health benefits.

## 1. Introduction

Due to the scientific evidence about the risks of sugary and energy drinks, in recent years, scientific societies and public demand have been providing a new definition of beverages. Functional drinks such as Kombucha tea are popular with consumers today because they provide an appropriate combination of vitamins, metabolites, proteins, fiber, and other essential nutrients along with maintaining an acceptable flavor. In the meantime, Kombucha tea becomes a very acceptable and popular beverage.

**Figure 1:**
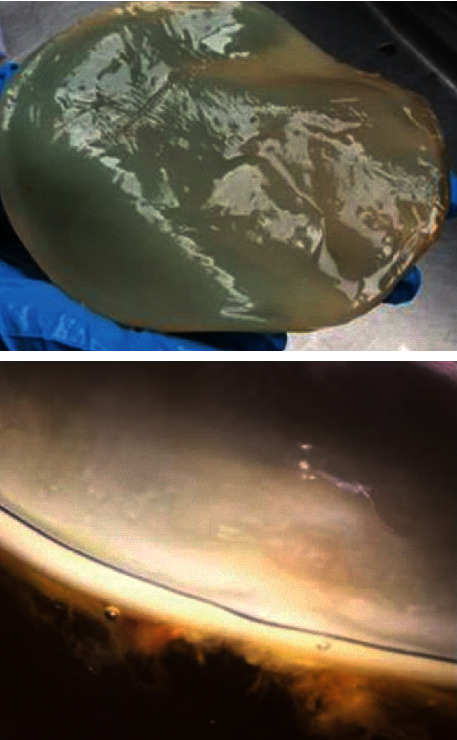
Kombucha SCOBY, which is fed by sucrose [3].

Kombucha beverage is a naturally fermented beverage that is acquired from sugary tea [1], with a dependent Symbiotic Culture of Bacterium and Yeast (SCOBY) [2] via a fermentation process usually lasting for 7–10 days. A biofilm of SCOBY, which is more similar to a mushroom cap, may act as a starter for any fermentation. The colony is created from *Rhodospirillales cluster* (*Acetobacter xylinum* bacteria and *Gluconobacter*), ascomycetous, and *Saccharomyces ludwigii*. Carboxylic acid and carbonic gas are accomplished, due to the fermentation of polysaccharides to simpler carbohydrates [3]. The SCOBY contains various yeasts like *Pichia* spp. [4, 5], *genus Saccharomyces* sp. [5], *Torulopsis* sp. [5], *Zygosaccharomyces bailii* [4, 6], *Brettanomyces* sp. [5], *Zygosaccharomyces kombuchaensis* [5], and also many various acetic acid bacteria like *Acetobacter pasteurianus* [4, 5], *Acetobacter xylinum* [4, 6], *Gluconobacter oxydans*, and *Acetobacter carboxylic acid* [4, 5]. The results have shown that, after the fermentation process, Kombucha contains different chemical components [5, 7] such as metallic elements (e.g., Fe, Mn, Ni, Cu, and Zn); carbon dioxide; organic food acids; polyphenols; many water-soluble vitamins like vitamin C; amino acids such as lysine; fiber; sugars; antibiotic substances; different types of vitamin B; hydrolytic enzymes; and ethanol [8]. This SCOBY steadily grows and increases its thickness over time (Figure 1).

**Figure 2:**
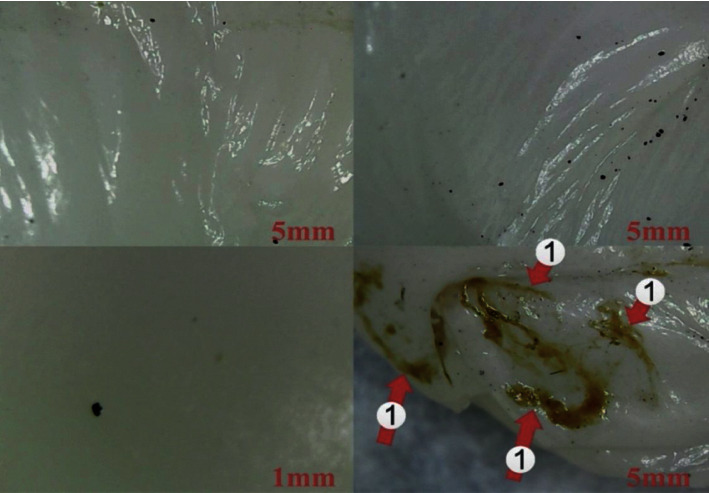
Optical microscopic images of Kombucha SCOBY (number 1 demonstrates the dead Kombucha bacteria) [9].

Figure 2 illustrated the optical microscopic images of Kombucha SCOBY [9]. Unfortunately, there have not been enough accessible clinical studies to prove Kombucha's benefits for human health yet [10]. Several benefits, such as antioxidant activity and anti-inflammatory potential, make Kombucha popular as a functional beverage or food [11]. Moreover, Kombucha can reduce blood pressure, and also it can inhibit cancer growth [12, 13]. Finally, it is necessary to say that Kombucha can be used as a robust material for improving the immune system and function of the liver and gastrointestinal [14]. This wide range of advantages has led to developing the practicality of Kombucha and investigating the role of the microbiome on health [15]. The liquid acid broth and a cellulosic pellicle are the main two components of Kombucha [16]. The liquid broth has various major and minor components like gluconic acid, acetic acid, carboxylic acid, glucuronic acid, and ethanol.

In addition to the mentioned parts, Kombucha has phenolic components that contain epicatechin gallate, epigallocatechin, catechin, epicatechin, and epigallocatechin gallate [17]. The presence of enzymes and vitamin B is well confirmed according to the results [18]. So the liquid broth has already been shown to have a significant impact on immune response and also thought to be a compelling factor for stomachic ulcers [19] as anticarcinogenic, antioxidant, and liver detoxification.

**Figure 3:**
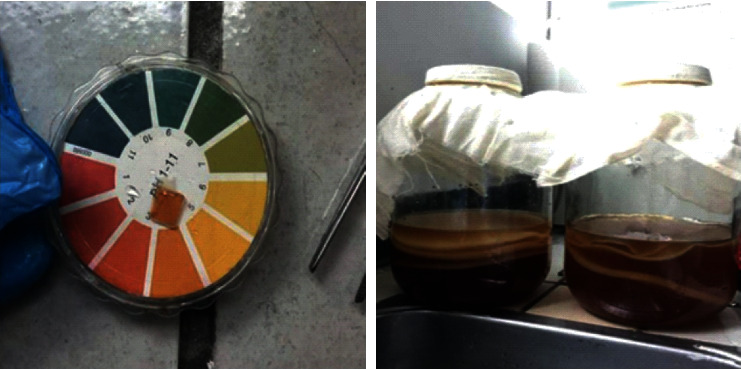
Kombucha acidic environment [3].

Many researchers have tried to analyze the antimicrobial functions of Kombucha; for instance, Steinkraus et al. in their valuable works proved that Kombucha has excellent antimicrobial activity against several microorganisms including *Staphylococcus aureus*, *Agrobacterium tumefaciens*, *E. coli*, and *Helicobacter pylori* that this good activity can be attributed to its acetic acid content [20, 21]. As shown in Figure 3, using the pH indicator, the pH quantity of 4 can be seen during the process. Our paper aims to summarize and describe Kombucha's properties toward biological and medical applications and also characterize opportunities for future research.

## 2. Chemical Composition

For more understanding of Kombucha's kinetics, we need to have a definitive and comprehensive study on its composition and properties. Several essential factors such as fermentation time, the tea and sugar concentration, the used temperature, and the inoculum supply may affect the composition and concentration of the metabolites [22]. The fermentation conditions should be controlled to get an excellent final product. However, Table 1 demonstrated some metabolites produced within the fermentation process. Glucuronic acid is a sugar acid derived from aldohexose in which the sixth atom oxidized to the carboxylic acid. The recent interest within the metabolism of glucuronic acid has arisen mainly as a result of the fact that the conjugation of glucuronic acid is a vital step in the metabolism of steroid hormones and adrenalin and also following the reports, glucuronic acid could be helpful in arthritis disease. The mechanism of the glucuronides formation has been principally determined in studies of drug metabolism and has been widely accepted as a detoxication method. Glucuronic acid is one of the components in Kombucha that is extremely impactful because of its drug metabolism [23]. Another component is ethanoic acid, which is a vital chemical and traditionally platform used as a food preservative [24]. It is a transparent, colourless, corrosive acid with a bitter pungent smell. Ethanoic acid is synthetically created by microorganism fermentation. The increasing significance associated with the high demand for ethanoic acid has promoted an interest in medicinal usage of this acid [25]. Indeed, ethanoic acid is the main product of Kombucha fermentation.

**Figure 4:**
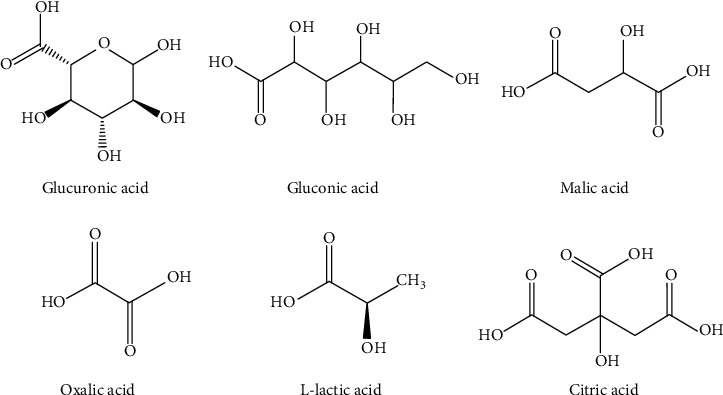
Several organic acids found in Kombucha as a result of the fermentation process [6].

The final concentrations of sugar in each fermentation process can vary, which indicates that various factors can regulate the effects, so the metabolism pathway is not usually equal [26]. Researchers have gotten many new records for the duration of the fermentation process; for example, Jayabalan et al. have gained the growing quantity of acetic acid for the period of the maximum fermentation attention of approximately 9.5 g/L after two weeks [27]. Preliminary sugar concentration and many different factors ought to without difficulty affect the concentration of each metabolite produced in Kombucha as well as the chemical composition (Figure 4).

**Figure 5:**
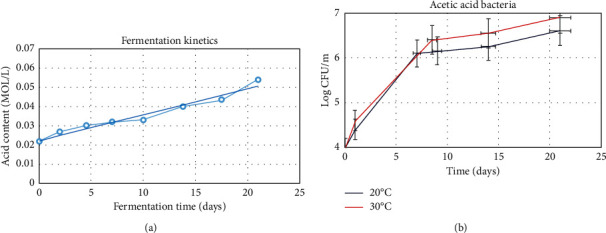
(a) Fermentation kinetics and (b) microbial loads (log CFU/ml) of acetic acid bacteria (AAB) in Kombucha.

When the fermentation improves [28], the yeast portion of this complex culture can decompose sucrose to glucose, fructose, and carbon dioxide; therefore, this performance leads to the release of the gas and carbonated appearance. Indeed the transformation process between glucose and gluconic acid toward fructose into acetic acid commonly is performed by the acetic acid bacteria. Besides, after producing ethanol by the yeast portion, ethanol can be oxidized into acetaldehyde using the bacterial counterpart of this colony [29]. During ethanol production, the yeast portion chooses fructose as the layer [30]. There is a dual relationship between acetic acid produced by the acetic acid bacteria and ethanol [31]. The acetic acid which is provided via the acetic acid bacteria can quickly motivate the ethanol's production via yeast; on the other hand, ethanol can simplify acetic acid production and the growth of acetic acid bacteria. Ethanol and acetic acid present in Kombucha could act as active antimicrobial agents against pathogenic bacteria, which will be discussed in detail in the following parts [30]. Scientists, in their exciting works, analyzed the acidic value of Kombucha for about three weeks to investigate and characterize the fermentation process kinetics. As demonstrated in Figure 5(a), titrimetric tests indicate that, with increasing fermentation time, acidity also increases by a rate of 1.5 mm per day [7]. Hence, the chemical compositions of Kombucha demonstrated that this valuable product possesses different applications, which are discussed in detail below.

## 3. Production of Kombucha

As stated in previous sections, Kombucha is composed of bacteria and yeast, which produces bacterial cellulose pellicles on the broth. A cellulose pellicle of Kombucha possesses a delicate structure with acceptable purity. It has been used in several forms like a gelling agent, stabilizer, emulsifier, and dispersing factor. Kombucha is receiving considerable attention and being extensively investigated as a novel type of framework due to its high biocompatibility and pliability [11]. Also, it has been reported that this product has excellent water holding capacity and high durability with a satisfactory degree of polymerization [32]. The role of Kombucha in tissue engineering as artificial skin for wounds or scaffold of blood vessels and cartilages has been highlighted during the last years. As Kombucha is nonallergic and nontoxic, it can be utilized in the food industry as dietary fiber and additive [33]. Considering these properties and applications, it is highly prerequisite and necessary to investigate the fermentation methods of this product. Kombucha can be prepared through previously fermented broth or fermenting tea with a pellicle, and the favorable temperature of the substrate is 20 to 28°C (Under aerobic conditions). Based on the previous literature, it can be stated that the fermentation time can differ up to about two months. Due to differentiation in the duration of fermentation, various tastes and flavors are provided. For example, Kombucha, with long fermentation (60 days), has a slight vinegar taste [26]. The presence of yeasts in Kombucha verifies the hydrolysis of sucrose to other monosaccharides, and in the next step, yeasts can metabolize them to ethanol. Then ethanol is oxidized to acetic acid by acetic acid bacteria. As acetic acid bacteria do not have extracellular hydrolysis enzymes, they are not able to uptake sucrose alone [34]. Recently, the researchers have claimed that the fermentation of Kombucha is not restricted to using sweetened black tea. They reported that several substrates like wine, herbal tea, green tea, or fruit drink could be used in the fermentation of Kombucha. In some cases, these proposed that substrates are more efficient than conventional tea. The usage of other substrates like thyme, peppermint, and Jerusalem artichoke tuber in Kombucha fermentation was investigated. In terms of Jerusalem artichoke tuber, it has been accepted that the obtained Kombucha is an excellent dietetic product due to the presence of inulooligosaccharides in its structure [35].

The use of alternative raw materials (e.g., coffeeberry, leaves, fruits, milk, vegetables, by-products, and wastes) for the fermentation process of Kombucha has been proposed by researchers. For example, by using by-products and wastes in the fermentation process, not only can the time and cost of fermentation be reduced, but also more significant antibacterial and antifungal activity of the final product can be achieved. Similarly, tea with some additives as the raw alternative can enhance the antioxidant and anticancer performance of Kombucha [20]. In the past, a solid-state fermentation method was applied by researchers to produce Kombucha. However, this fermentation method has several limitations like expensive equipment, low yield, and complicated process. Hence, the submerged fermentation method can be a promising candidate to surmount the mentioned limitations. Several factors like duration of fermentation, substrate type, pH, and temperature can influence the final product; however, due to the comprehensive nature of the available data and some limitations on the length of this section, only essential points are explained. The optimum temperature was maintained throughout the fermentation process, which leads to better enzyme activity and microbial growth and improvement of the fermentation benefits. Moreover, temperature variations can influence the antioxidant effect in substances with the plant sources [32], for instance, the production of the compounds that contain phenol. The temperature values of the Kombucha fermentation process can commonly be considered from 20°C to 30°C. Some researchers in their exciting works investigated the microbial loads (log CFU/ml) of acetic acid bacteria (AAB) in Kombucha for two different temperatures (Figure 5(b)) [36].

For example, in Vitas et al.'s study, they performed the fermentation process based on the temperature variations using optimization designs. They claimed that the temperature could be considered as the most critical factor during the fermentation process. Therefore, in the temperature ranges between 37°C and 42°C, the highest antioxidant performance can be achieved. Moreover, researchers concluded that, with increasing temperature, the amount of vitamin C and generated acids, as well as metabolites, were increased in obtained samples. The fermentation of Kombucha lasts commonly in the range of 7 to 60 days, and biological effects were enhanced within this process. However, desirable results have been obtained in about 15 days [37]. Although most of the achieved antioxidant activities have been enhanced with incubation time, the long-term fermentation is not proposed due to the accumulation of organic acids, which could be harmful to direct usage [38]. Moreover, the produced CO_2_ can start to be accumulated at the junction of the broth and biofilm. This may close the transfer of the nutrients and leads to an unusable environment [37]. Also, the period of fermentation can be selected depending on the expected features. Reiss informed that a fruit-like refreshing beverage was attained in the ranges of 6 to 10 days of the fermentation period, contrary to the vinegar taste that is obtained in the higher duration. Based on the Food and Drug Administration model food code for Kombucha brewing considering human consumption, more than 10 days of fermentation is not proposed [39]. Coton et al. investigated the evolution process of microbial populations from the industrial Kombucha tea in the duration of 0, 2, 4, and 8 days [39].

**Figure 6:**
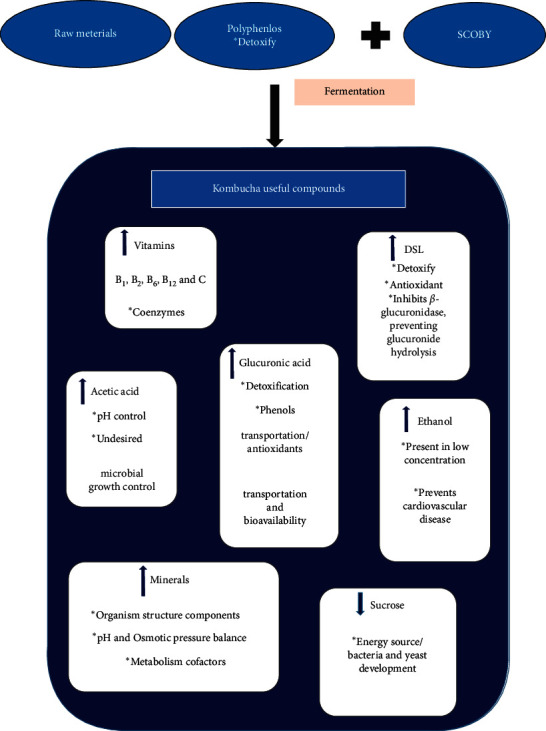
Kombucha beneficial compounds.

The results exhibited that AAB, more abundant in the biofilm than in the liquid broth, reached equilibrium after eight days, contrary to yeast species that seemed to be stable completely in both phases throughout the fermentation process. Some researchers estimated the amount of polyphenol and the antioxidant activity of Kombucha during its fermentation period (0, 7, 14, and 21 days) and demonstrated a high tendency to increase especially after the 7th day, which can be because of the more microbial variety obtained by that time [40]. The presence and values of the chemical compositions largely depend on applied symbiotic microorganisms for Kombucha fermentation [41–43], as well as the temperature and time of fermentation and type of applied tea and sucrose value, in addition to the analysis methods applied for quantification. Advantageous metabolites produced in Kombucha are presented in Figure 6 [14].

## 4. Pharmaceutical Effects of Kombucha

As a perpetual advantage, Kombucha vendors and drinkers have always claimed that this miraculous tea has many beneficial effects on human health. Nevertheless, many of these claims have not been substantiated by rigorous scientific evidence based on human trials, and there are only a few experimental documents to prove it. There are some clinical trial and nonhuman experiments regarding anticancer, antioxidant, antibacterial, antifungal, hepatoprotective, and some other health beneficial effects for Kombucha beverages which are reviewed in this section.

### 4.1. Anticancer Activity

**Figure 7:**
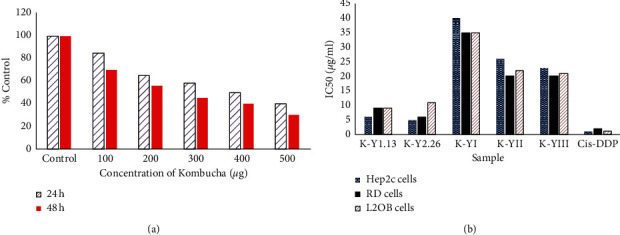
(a) Effects of Kombucha on PC-3 cell viability. (b) MTT assay graph of yarrow Kombucha beverages.

*Cancer* is one of the most important causes of today's worldwide death. Therefore, to reduce the mortality rate, anticancer drugs with less destructive properties can be beneficial [44]. Many compounds were introduced as anticancer agents until now, with lower side effects [45, 46], and clinical studies have contended that Kombucha has anticancer activities as well. [30] The Russian Academy of Sciences in Moscow and the Central Oncological Research Unit in Russia have done many investigations on Kombucha, and they gained that this valuable beverage can inhibit the cancer cells [30]. Scientists have explored any possible pathway to determine the anticancer effects of Kombucha, and finally, some of them claimed that the presence of tea polyphenols and the secondary metabolites produced during the fermentation process has the most impact [8, 47]. Based on the results, it can be said that the tea polyphenols which are present in Kombucha can prevent gene alteration and also it can inhibit the propagation of cancer cells and make cancer cell apoptosis, and the capability to complete metastasis has been determined as conceivable functions for the anticancer features. Indeed, using Kombucha tea can be an effective way to reequilibrate blood pH for cancer patients, whom their blood pH is 7.56 or more during illness. Moreover, a suitable solution for cancer patients who do not have enough L-lactic acid in their connective tissues is the consumption of Kombucha, because one of the products of the fermentation pathway of Kombucha is exact L-lactic acid [30]. Components like vitamin C, glucuronic acid, polyphenols, gluconic acid, and lactic acid which are playing the critical roles in Kombucha are capable of diminishing the incidence of stomach cancer [48]. Another indirect factor which is related to cancer can be D-saccharic acid-1,4-lactone (DSL) that was identified to be present in Kombucha, and the main activity of DSL is to inhibit the glucuronidase task [49]. Besides, glucuronidase can be utilized as a glucuronide hydrolyzer, and it can generate cancer-causing aglycones [50]. It was proven that presence of dimethyl-2-(2-hydroxy-2-methoxypropylidene) malonate and vitexin in the ethyl acetate fraction of Kombucha has demonstrated appropriate cytotoxic activities at a concentration of 100 *μ*g/mL [47] and the polyphenols in Kombucha tea have the same performance and both of them are reported as cancer prevention agent [49]. It is essential during the fermentation process to observe the health principles because pathogenic microorganisms are capable of infecting the whole beverage by the preparation process [51]. After investigating the effects of Kombucha on the human body, the US Food and Drug Administration confirmed that there is no harmful effect of this product on the body and on the other hand, a group of researchers examined the toxicity of Kombucha in mice for 3 months, and all the symptoms and results were reported. The anticancer advantages of Kombucha were considered as folklore, with no deep-rooted clinical backing. But, some researchers tried to regard Kombucha as a potent agent for renal and liver cancer cells therapies [47]. A general and appropriate assay for assessment of biomaterial toxicity is the MTT method. This assay is related to the metabolisms of cells and mitochondrial functions. Therefore, the cell viability of Kombucha-treated PC-3 cells was evaluated through the MTT method. Based on the results, it can be easily said that the IC 50 value of Kombucha (against PC-3 cells for 24 h treatment) was determined at approximately 400 *μ*g/ml. As indicated in Figure 7(a), with increasing the concentration of treatment in Kombucha, the percentage of cell viability diminished slowly [52].

Srihari et al. in their interesting study investigated the anticancer activity of Kombucha, and therefore they found that Kombucha beverage could be an active agent for preventing the growth of metastasis in prostate cancer.  Figure 7(b) illustrated the antiproliferative effect of yarrow Kombucha products. Indeed, three distinct cell lines such as cell line obtained from murine fibroblast (L2OB), cell line derived from human rhabdomyosarcoma (RD), and cell line derived from human cervix carcinoma Hep2c (HeLa) were utilized in order to examine the anticancer effect of Kombucha and as a result, all the materials exhibited antitumor effect. Kombucha inoculum, used in their study, was fermentation liquid of Kombucha on yarrow infusion and subcritical water extract, and yarrow was chosen for introducing a novel substrate for Kombucha fermentation and therefore the following Kombucha beverages were produced: K–Y1.13, K–Y2.26, K-YI, K-YII, and K-YIII (they are in different weight percent) [53]. It can be concluded that Kombucha is an anticancer agent; however, still clinical experiments and more *in vivo* investigations should be carried out to highlight the role of Kombucha as anticancer agent.

### 4.2. Antioxidant Activity

Antioxidant activity belongs to any substance that can hinder or delay oxidation of the substrate even in low concentrations [54]. Indeed, this activity can be extensively presented in three forms like inhibition properties of molecules, scavenging of prooxidant enzymes, and binding of prooxidant metals. So far, the effects of these antioxidant activities on several illnesses like diabetes and cancer were proven [37]. Removing free radicals produced in oxidation reactions is the first performance of the antioxidant activity. As we know, the generation of free radicals is not beneficial for the human body, because they are able to commence the multiple chain reactions and finally these reactions lead to harm cells or even death [54]. The balance between cellular ROS generation and antioxidant defence capacity determines the status of oxidative stress [55].

**Figure 8:**
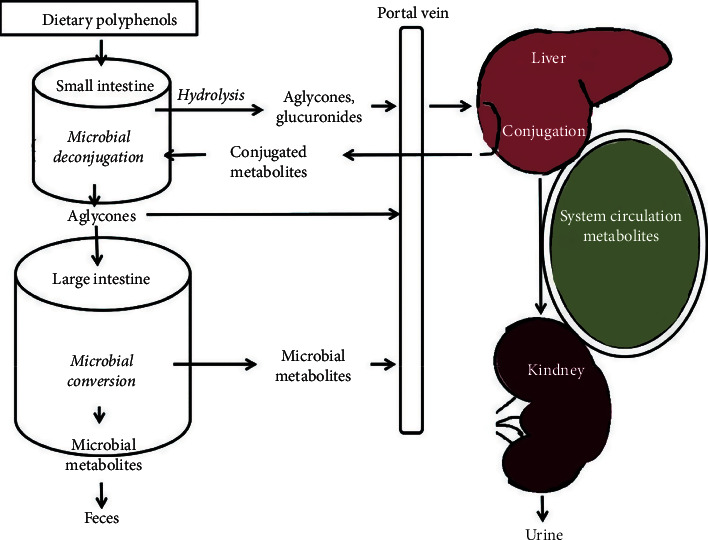
Metabolic fate of dietary polyphenols [61].

Although Kombucha's fermentation process does not possess a complicated pathway [56–58], several beneficial compounds of this product with radical inhibiting activities are founded as useful antioxidant agents. It can be said that catechins and polyphenols are two primary classes of compounds that are presented in Kombucha beverage and also they belong to flavanol category. Polyphenols are very wonderful compounds that have been found in Kombucha and can efficiently scavenge free radicals reactive oxygen species (ROS) which is the reason for introducing Kombucha as a potent antioxidant agent. Polyphenols indicate different structures, of which tannins, flavonoids, phenolic acids, and stilbenes are regarded as the primary structures of dietary polyphenols. Document from various investigations has provided the relations between the polyphenols' intake and reduced occurrence of several human illnesses and also confirmed their function in the inhibition of cancer, neurodegenerative disorders, obesity, and cardiovascular. Specific polyphenols can be presented in the shape of polymers, glycosides, or esters; therefore, they are not able to intake in their general forms. First, they should be hydrolyzed using intestinal enzymes or even by the colonic microflora before absorption. Indeed, polyphenols tolerate different conjugation mechanisms like sulfation, methylation, and glucuronidation initially in the intestinal cells and then within the liver [59]. As shown in Figure 8, the unabsorbed polyphenols are biotransformed into a series of low-molecular-weight phenolic metabolites within the gut. These mentioned metabolites should possess better absorption activity than their previous precursors and can exert positive features in the human digestive system (local effects) and, after being absorbed, in organs and tissues (systemic effects) [60].

It has been proven that the presence of complex phenolic compounds in an acidic environment or even the release of various species of enzymes by fungi and bacteria can lead to the splitting of large and complex molecules into smaller and simpler molecules, which leads to an increase in phenolic compounds in Kombucha [62]. Researchers have measured an antioxidant activity of Kombucha (with an infusion of rooibos leaves) through two different methods like 2,2-diphenyl-1-picrylhydrazyl radical (DPPH) and ferric ion reducing power (FRAP) [38]. In Kombucha, which contains rooibos leaves, there are no catechins, and this may lead to having lower antioxidant activity in comparison with Kombucha lonely. On the other hand, the researchers claimed that Kombucha with rooibos leaves possesses other components like orientin, aspalathin, and rutin (mainly) and, at lower concentrations, isoquercitrin, hyperoxide, and isovitexin, which all of them have antioxidant effects. Another group of researchers investigated the fermentation time and Kombucha origins on its antioxidant activities by in vitro free radical scavenging assays and they concluded that, with increasing the fermentation time, the antioxidant activities increased. This activity has a direct relationship with origin materials and culture time, each of which, in turn, identifies the type of metabolites itself. Greenwalt et al. claimed that, due to the agglomeration of some organic acids that may be detrimental for direct usage, the prolonged fermentation process is not a proper pathway, although antioxidant activities of Kombucha exhibited the time-dependent profiles [4]. To clarify the metabolic direction in the fermentation of Kombucha, the determination of vital extracellular enzymes, as well as potent metabolites, is required.

### 4.3. Antimicrobial and Antifungal Activity

Since the investigation of antibacterial and antifungal properties is a vital issue in biochemistry and pharmacy, so far a significant group of researchers has insisted on this, and therefore the preliminary element of their articles was to determine these properties [63, 64]. It is proven that Kombucha has a supreme antimicrobial effect on a wide range of microorganisms [65] and so far many evaluations have been carried out to demonstrate the inhibitory performance against plenty of microorganisms including Gram-negative and Gram-positive bacteria [30]. In fact, Kombucha is able to prevent the growth of an extensive range of microorganisms [66] like *Pseudomonas aeruginosa, Agrobacterium tumefaciens, Helicobacter pylori* (the causative organism of peptic ulcers), *Enterobacter cloacae, Salmonella enteritidis, Escherichia coli* (the causative organism of common diarrhea), *Yersinia enterocolitica, Candida albicans, Shigella sonnei, Campylobacter jejuni,* and *Staphylococcus aureus*. Perhaps this proper antimicrobial effect is due to the presence of acetic acid in this SCOBY, and also low levels of pH can be beneficial [67]. Also, the proteins produced during the fermentation process and the catechins and some of the components shown in Figure 8 can be considered as critical factors for the improvement of antibacterial properties. It is accepted that both catechins and acetic acid found in Kombucha have bactericidal properties and also it was reported that Kombucha has antibiotic substances that lead to an increase in the antimicrobial performance. The acetic acid in Kombucha not only improves the antimicrobial properties but also can enhance antifungal properties as is proved. Recently, due to the excellent antimicrobial activity of Kombucha, it can be extensively utilized as an agent to reduce the pathogens associated with human illnesses. The scientists reported that the preparation process and fermentation time and also the origins could affect the antimicrobial and antifungal activity of Kombucha. To improve the antibacterial and antifungal activities of some substances [68], Kombucha can be utilized as an additive part and get better results. For example, Ashrafi et al. investigated the antimicrobial effects of chitosan/Kombucha films against *Staphylococcus aureus* and *Escherichia coli*. They claimed that the inhibition properties of chitosan/Kombucha films mainly depend on the Kombucha concentrations and any films which contain a higher level of Kombucha have better antimicrobial activity and antioxidant effect as shown in Table 2 [69]. Another group of researchers investigated the antimicrobial activity of yarrow Kombucha beverages. Their examination included evaluation of the samples against six diverse bacteria (*Escherichia coli, Proteus mirabilis, Proteus vulgaris, Staphylococcus aureus,Klebsiela pneumoniae*, and *Bacillus subtilis*) and two yeast (*Aspergillus niger* and *Candida albicans*). The minimum inhibitory concentration (MIC) results [63] of antimicrobial activity for Kombucha beverages are summarized in Table 3. Since MIC values were in the limitation of 9.765 *μ*g/mL to 312.5 *μ*g/mL, all the samples could be considered as antimicrobial agents in comparison to the standard antibiotics Amracin (for bacteria) and Nystatin (for yeasts) [53]. According to literature about antibacterial and antifungal performances of Kombucha, this valuable product can be applied in amelioration of infection diseases.

### 4.4. Hepatoprotective Effects

The capability to hamper the damage happening to the liver through toxic material is hepatoprotection. Based on results that have been done on animal models and cell lines, it is proven that Kombucha broth has hepatoprotective performance against different environmental contaminants and as we know, many environmental contaminants can lead to hepatotoxicity and also they can cause damaging to the liver [8]. Several clinical investigations were performed to evaluate the capability of Kombucha to helpfully reduce the physiological alterations [70] which are because of many hepatotoxicity-causing agents like cadmium chloride, acetaminophen, aflatoxin B1, and tert-butyl hydroperoxide. In an extensive study on mice, Kombucha was introduced as an inhibitor for carbon tetrachloride (CCl_4_) activity. As we know, CCl_4_ is a xenobiotic substance which can lead to the peroxidation of lipids and therefore makes free radicals CCl_3_^–^ and finally in the normal process destroys the liver [71]. How Kombucha has the ability to induce oxidative stress in Albino mice using chromate (VI) was the main aim of several investigations [72]. Indeed, investigations have also accomplished to determine the preservative activities of Kombucha toward thioacetamide-induced hepatotoxicity. Furthermore, it is proven that Kombucha is able to prevent the death of apoptotic cell related to the hepatocytes. Based on histological tests which have been carried out on alloxan-induced diabetes mice, it is accepted that Kombucha can be utilized as a protective liver-kidney agent for rats which have been a diet involving Kombucha broth [73]. So far, some scientists have performed many investigations toward hepatoprotective activities of Kombucha in mice and they concluded that this valuable activity can be supported by the devaluation in the function of alanine transaminase, aspartate transaminase, gamma-glutamyl transpeptidase in the plasma, and the concentrations of the creatinine urea. As a point, it can be mentioned that the antioxidant activity of Kombucha can be enhanced by manipulating several factors like a metabolic pathway, time, and temperature which were previously discussed.

### 4.5. Other Beneficial Effects

There are plenty of declarations about the uses and benefits of Kombucha beverage for health. The pathway of eliminating toxic substances from the organism's body is called the detoxification process. This complex action can be in two forms of medicinal or physiological. As we know, the liver plays a vital role in the body and one of the main tasks of the liver is detoxification. Detoxification function can be effective in cancer prevention action and also facilitates the preservation of the liver. The bacterial acids, enzymes, and the other secondary metabolites generated by microbes during fermentation which were carried out in the preparation of Kombucha enabled body detoxification [30]. Meanwhile, most of the bacterial acids and enzymes presented in Kombucha resemble the chemical compounds produced through the body for the purpose of the detoxification action. Therefore, incorporating the Kombucha tea into one's diet may lead to the reduction of liver detoxification. Several scientific reports have revealed that this ability mainly belongs to the capacity of binding glucuronic acid to toxic substances which arrived in the human body and also the capability to enhance excretion of these toxic molecules from the body using the cooperation of intestines and kidney [27]. Other than supporting the detoxification of the liver, consumption of Kombucha is also known to help excrete heavy metal substances and environmental pollutants from the human body through the kidneys [74]. It is also useful for biotransformation of indigenous metabolites, for example, bilirubin and excess of steroid hormones [62]. The noxious materials removal action of Kombucha tea helps to obtain relief from gout, rheumatism, arthritis, and kidney stones which are accompanied by the accumulation of toxins in the body [30]. Probiotics as living microorganisms can result in benefits of health when administrated in sufficient amounts. Mostly, the bacterial components of a probiotic material come from Bifidobacterium or Lactobacillus or a combination of these two strains. In this case, there can be a few usual yeast types such as *S. cerevisiae* and *Saccharomyces boulardii* in the mixture as well [75].

Probiotic microbes are known for their vital role in human health. These microorganisms create a balance in normalizing processes in the gut, intestinal microbiota, and boosting the immune system [33, 76]. Most of the studies have asserted that Kombucha not only is a probiotic but also acts as being symbiotic, a mixture of probiotics and prebiotics [30, 77]. A prebiotic selectively helps the activity and growth of the consortium of beneficial microbes present in the human gut [77]. The yeast and bacteria in this beverage operate as probiotics, and the present microcellulose can help in the growth of the useful microbes in the intestine [75]. One of the health benefits related to this beverage in Nossa Senhora da Conceição Hospital from Lagarto, SE, Brazil, is utilizing the present microbial mat in the fermentation for producing artificial skin [78]. Some of the researchers have used this skin for accelerating the healing pathway and adhering it to open injuries as an antiseptic, the so-called Bioskin [78]. The bacterial cellulose which is obtained in the course of the Kombucha fermentation shows many useful applications in the fields like biopharmaceuticals and food. The tendency of bacterial cellulose to be used in these fields is owing to the high purity and the unique physicochemical activities which are present in the fermented beverage.

Moreover, this bacterial cellulose is preferred in such a case where plant-based cellulose cannot be applied [79]. Oxalic acid is also a useful by-product which can be used in adenosine triphosphate (ATP) production [8] and in the food industries. Bacteria-based cellulose is applied as thickeners, food matrices, stabilizes, dietary fibers, and binders [79]. The present bacteria in the Kombucha mat generate gluconic acid by the breakdown of caprylic acid which can inhibit certain types of yeast-based infections and candidiasis [27]. Lactic acid as one of the produced organic acids in the Kombucha tea fermentation can be able to control the blood circulation and assists to hinder constipation [30]. One of the Butyric acid's tasks is to protect the cellular membrane of humans where it incorporates gluconic acid and reinforces the gut walls in a situation like candidiasis [8]. Also, based on several biochemical and histopathological investigations, Kombucha could be an advantageous factor for treating gastric ulcers [19]. The researchers claimed that Kombucha has the ability to inhibit *α*-amylase and lipase enzyme performance in the pancreas and even in plasma and also better suppression of increased blood glucose levels in rats and finally it is proven that Kombucha has effective hypoglycemic and antilipidemic activities [73].

## 5. Adverse Effects of Kombucha

Kombucha consumption can be harmful in several cases where it is prepared incorrectly, and further consumption should be prevented in individuals with preexisting conditions which can lead to metabolic acidosis effects. After consuming certain Kombucha products, some side effects such as dizziness and nausea have been reported, and also some allergic reactions, jaundice, and head/neck pain have occurred in several patients. Another point is that the consumption of Kombucha should be prevented in lactating or pregnant women [80].

## 6. Conclusions and Perspectives

So far, much effort has been devoted to investigating the different activities of Kombucha and it is proposed that Kombucha has the ability to mitigate arthritis, support cholesterol levels in good health, recover digestion, decrease blood pressure, and heal peptic ulcer illness, diabetes, and asthma and also it can be utilized in a wide range of cancers like prostate and liver. It is also claimed to possess hepatoprotective activities, encourage weight loss, provide preservation against different pathogenic microorganisms, and relieve hemorrhoids. The liver detoxifying activities of Kombucha have belonged to the effects on the glucuronidation process while the catechins and acetic acid present in Kombucha are thought to be responsible for antifungal and antimicrobial performance against different microorganisms.

Despite traditional beliefs and several articles, these claimed effects still require further studies and scientific validations. SCOBY, as the primary fermentation inoculum in Kombucha beverages, has not been well understood to date, and its microbial content still needs further studies. It is suggested to continually prepare and improve the new starter inoculum to identify better and characterize the microbial content of this fermented drink. This preparation ensures that Kombucha tea is produced in a good quality.

Besides, with the growing popularity and consumption of this functional drink, concerns about its potential risks and safety issues have also increased. Various pathogens found in Kombucha beverages are mainly due to contamination of unreliable raw materials, vessels, and packages, as well as insanitary producing environments during the fermentation process, which sometimes provide dangerous toxic metabolites such as mycotoxins. Also, antinutritional components such as cyanogenic glycosides, phytates, tannins, and protein inhibitors may be formed during the unhealthy fermentation process. The level of alcohol content of Kombucha is another problem that needs to be addressed. Therefore, health and nutrition organizations must set some regulations about the standardization of Kombucha production methods, identification of raw materials, and quality control soon. Although some countries currently have such regulations, they do not appear to be sufficient for such a high volume product and require a thorough revision of the industrial product manufacturing process and precise safety guidelines.

Given the properties of Kombucha, it seems that combining this product with other materials can yield novel beneficial compounds that, in addition to their excellent performance, can be very useful in medical science and supplementation. Several approaches have been suggested for such an assembly; however, there are many other factors during these pathways, which are still incomprehensible or unexplored.

## Figures and Tables

**Table 1:** General chemical composition of Kombucha [11].

	Compound	Average composition	Initial sucrose	Fermentation time (days)
General composites	Ethanol	5.5 g/L	100 g/L	20
	Proteins	3 mg/mL	100 g/L	12
	Polyphenols	7.8 mm GAE	100 g/L	15

Organic acids	Acetic acid	5.6 g/L	70 g/L	15
	Acetic acid	8.36 g/L	100 g/L	18
	Acetic acid	11 g/L	100 g/L	30
	Gluconic acid	39 g/L	100 g/L	60
	Glucuronic acid	0.0160 g/L	70 g/L	21
	Lactic acid	0.18 g/L	100 g/L	18

Minerals	Cu, Fe, Mn, Ni, and Zn	0.1 to 0.4 *μ*g/mL	70 g/L	15

Vitamins	Vitamin B_1_	0.74 mg/mL	70 g/L	15
	Vitamin B_2_	8 mg/100 mL	70 g/L	10
	Vitamin B_6_	0.52 mg/mL	70 g/L	15
	Vitamin B_12_	0.84 mg/mL	70 g/L	15
	Vitamin C	25 mg/L	70 g/L	10

Anions	F^−^, CI^−^, Br ^−^, I^−^, NO^3−^, HPO^4−^, and SO^4−^	0.04 to 3.20 mg/g	100 g/L	7

**Table 2:** Antimicrobial activity and antioxidant activity of chitosan/KT films [69].

Film samples	DPPH radical scavenging activity (%)	Inhibitory zone (mm), *Staphylococcus aureus*	Inhibitory zone (mm), *Escherichia coli*
CH	6.19 ± 0.57	8.66 ± 0.33	6.21 ± 0.51
CH+1%KT	29.15 ± 1.21	13.20 ± 0.50	15.33 ± 0.33
CH+2%KT	44.56 ± 0.37	15.21 ± 0.00	20.11 ± 0.05
CH+3%KT	59.20 ± 1.21	17.66 ± 0.88	20.11 ± 0.05

**Table 3:** Antimicrobial activity of yarrow Kombucha beverages [53].

Microbial species	MIC values (*μ*g/mL)						
	K–Y1.13	K–Y2.26	K-YI	K-YII	K-YIII	A (Amracin)	N (Nystatin)
*Aspergillus niger*	39.10	156.25	78.13	39.10	312.50	—	0.97
*Candida albicans*	78.13	156.40	312.50	39.10	39.10	—	1.95
*Bacillus subtilis*	39.10	19.53	78.13	156.25	9.77	0.24	—
*Proteus mirabilis*	156.25	156.24	39.10	156.40	78.13	0.49	—
*Proteus vulgaris*	78.13	78.13	312.50	156.25	156.40	0.49	—
*Escherichia coli*	39.10	78.25	78.13	134.60	312.50	0.97	—
*Klebsiella pneumoniae*	19.53	19.53	312.50	156.25	78.13	0.49	—
*Staphylococcus aureus*	78.13	78.20	312.50	78.13	78.25	0.97	—

## Data Availability

All data supporting the findings of this study are available within the article.

## Abbreviations

SCOBY: Symbiotic culture of bacterium and yeast
DMI: 3-Dimethyl-2-imidazolidinone
GAA: Glacial ethanoic acid
TP: Thin prep
hrHPV: Human recombinant human papillomavirus
CAPD: Continuous ambulatory peritoneal dialysis
LDH: Lactate dehydrogenase
TCA cycle: Tricarboxylic acid cycle
MCT: Monocarboxylate transporters
PLA: Polylactic acid
PHAs: Polyhydroxyalkanoates
PEG: Polyethylene glycol
PCL: Poly-e-caprolactone
DSL: D-Saccharic acid 1,4-lactone
MTT: 3-(4,5-Dimethylthiazol-2-yl)-2,5-diphenyltetrazolium bromide
RD: Rhabdomyosarcoma
ROS: Reactive oxygen species
DPPH: 2,2-Diphenyl-1-picrylhydrazyl radical
FRAP: Ferric ion reducing power
MIC: Minimum inhibitory concentration
CCl4: Carbon tetrachloride
ATP: Adenosine triphosphate.
